# Acute Pulmonary Edema in COVID-19: Clinical Predictors, Long-Term Pulmonary Sequelae, and Mortality in a Romanian Cohort Study

**DOI:** 10.3390/jcm14228188

**Published:** 2025-11-18

**Authors:** Diana-Maria Mateescu, Dragos-Mihai Gavrilescu, Andrei Marginean, Ioana-Georgiana Cotet, Elena-Cristina Guse, Camelia-Oana Muresan, Ana-Olivia Toma, Stela Iurciuc, Adrian-Cosmin Ilie, Alexandra Enache

**Affiliations:** 1 Doctoral School, Department of General Medicine, “Victor Babes” University of Medicine and Pharmacy, Eftimie Murgu Square 2, 300041 Timisoara, Romania; 2 Department of Orthodontics, Dental District, Strada Zăgazului Nr. 3, ONE Floreasca Vista, Sector 1, 014261 Bucharest, Romania; 3 Department of Surgery, “Dr. Victor Popescu” Emergency Military Hospital, 9 Gheorghe Lazăr Street, 300080 Timișoara, Romania; 4 Centre of Molecular Research in Nephrology and Vascular Disease, “Victor Babes” University of Medicine and Pharmacy, 300041 Timisoara, Romania; 5 Legal Medicine, Timisoara Institute of Legal Medicine, 300041 Timisoara, Romania; enache.alexandra@umft.ro; 6 Ethics and Human Identification Research Center, “Victor Babes” University of Medicine and Pharmacy, Eftimie Murgu Square 2, 300041 Timisoara, Romania; 7 Discipline of Forensic Medicine, Bioethics, Deontology, and Medical Law, Department of Neuroscience, “Victor Babes” University of Medicine and Pharmacy, Eftimie Murgu Square 2, 300041 Timisoara, Romania; 8 Discipline of Dermatology, “Victor Babes” University of Medicine and Pharmacy, Eftimie Murgu Square 2, 300041 Timisoara, Romania; 9 Cardiology Department, “Victor Babes” University of Medicine and Pharmacy, Eftimie Murgu Square 2, 300041 Timisoara, Romania; 10 Department of Public Health and Sanitary Management, “Victor Babes” University of Medicine and Pharmacy, Eftimie Murgu Square 2, 300041 Timisoara, Romania

**Keywords:** COVID-19, acute pulmonary edema, NT-proBNP, IL-6, troponin, endothelial dysfunction, fibrosis, long-term pulmonary sequelae, cardio-inflammatory phenotype, biomarker-guided management

## Abstract

**Background/Objectives:** Acute pulmonary edema (APE) has emerged as an overlooked but life-threatening manifestation of COVID-19, reflecting the intersection of inflammatory, endothelial, and cardiac injury pathways. This study aimed to determine the incidence, independent predictors, and long-term pulmonary sequelae of APE in hospitalized COVID-19 patients. **Materials and Methods:** We conducted a retrospective cohort study including 127 consecutively admitted adults with confirmed SARS-CoV-2 infection at a tertiary-care center in Romania. Demographic, clinical, biochemical, and imaging data were analyzed. Multivariate logistic regression identified independent predictors of APE and in-hospital mortality, while three-month follow-up assessed pulmonary recovery and biomarker dynamics. **Results:** APE occurred in 36.2% of patients and was associated with a four-fold increase in in-hospital mortality (43.5% vs. 12.3%, *p* < 0.001). Elevated NT-proBNP, troponin I, and IL-6 levels independently predicted both APE occurrence and mortality. APE patients required more frequent ICU admission (52.2% vs. 18.5%, *p* < 0.001) and had longer hospital stays. At three months, 39% of APE survivors exhibited fibrotic CT changes, and 37% had restrictive ventilatory defects, correlating with persistently increased NT-proBNP and IL-6 concentrations. **Conclusions:** Acute pulmonary edema delineates a distinct cardio-inflammatory phenotype of COVID-19, driven by endothelial dysfunction and biomarker-elevated cardiac stress. Early biomarker-guided fluid management and structured multidisciplinary follow-up may mitigate both acute mortality and chronic pulmonary sequelae in post-COVID populations.

## 1. Introduction

Severe acute respiratory syndrome coronavirus 2 (SARS-CoV-2) infection primarily targets the respiratory tract, but systemic endothelial and inflammatory injury frequently extend beyond the lungs, leading to multisystem involvement. Early pathophysiological diagnosis of APE in COVID-19 is critical, as it represents a distinct entity from classic acute respiratory distress syndrome (ARDS), driven by endothelial dysfunction and cardiac stress. Biomarker-based prediction, including elevated NT-proBNP, troponin, and IL-6, can facilitate timely intervention. Additionally, the alveolar–capillary gradient has emerged as a promising tool for early detection of pulmonary vascular permeability changes in COVID-19, aiding in differentiating APE from ARDS and guiding fluid management strategies [1]. This approach enhances prognostic accuracy and supports personalized care in high-risk patients. Among the most severe manifestations, acute pulmonary edema (APE) has emerged as a critical clinical entity often underrecognized in early triage settings associated with acute respiratory distress syndrome (ARDS), cytokine storm, and myocardial dysfunction in COVID-19 patients [2,3,4]. The overlapping pathophysiological mechanisms between cardiogenic and non-cardiogenic pulmonary edema complicate differential diagnosis and management, particularly in patients with pre-existing cardiovascular disease.

The pathogenesis of APE in COVID-19 is multifactorial. The viral spike protein binds to angiotensin-converting enzyme 2 (ACE2) receptors expressed on alveolar epithelial and endothelial cells, resulting in increased capillary permeability, fluid extravasation, and alveolar flooding [5,6]. Simultaneously, hyperinflammation, cytokine release (notably IL-6, TNF-α, and IL-1β), and microvascular thrombosis amplify pulmonary congestion and impair gas exchange [7]. Cardiogenic factors such as acute left ventricular dysfunction, stress-induced cardiomyopathy, and myocarditis further exacerbate fluid accumulation in the alveolar space [8]. Elevated biomarkers—including NT-proBNP, troponin, and IL-6—have been consistently associated with APE and poor outcomes in COVID-19 cohorts [9,10]. The convergence of endothelial inflammation and cardiac stress defines a unique cardio-pulmonary phenotype that differs mechanistically from classical ARDS.

While pulmonary edema was initially attributed mainly to ARDS or fluid overload, emerging evidence suggests that COVID-19-related APE represents a distinct pathophysiological subset marked by diffuse alveolar damage, endothelial barrier disruption, and impaired lymphatic drainage [11]. Clinically, APE manifests with sudden dyspnea, hypoxemia refractory to oxygen therapy, bilateral basal crackles, and radiographic evidence of alveolar infiltrates. Differentiating APE from pneumonia or ARDS often requires integration of imaging, echocardiographic findings, and response to diuretics.

The incidence of APE among hospitalized COVID-19 patients varies between 3% and 12%, depending on case definition and population characteristics [12]. Risk factors include advanced age, hypertension, diabetes mellitus, obesity, chronic kidney disease, and pre-existing heart failure [13]. However, data on APE-specific mortality and long-term pulmonary sequelae remain limited. Persistent dyspnea, reduced lung diffusion capacity (DLCO), and fibrotic changes have been documented months after acute illness, yet the contribution of APE to these outcomes is not fully understood [14,15].

Romania has experienced multiple pandemic waves with heterogeneous vaccination uptake and high cardiovascular comorbidity burden. Understanding the determinants, clinical course, and long-term outcomes of APE in Romanian COVID-19 patients could provide valuable insight into post-acute sequelae and mortality risk in Eastern European populations, which remain underrepresented in current literature [16].

Therefore, the present study aimed to evaluate the clinical predictors, biomarkers, and outcomes associated with acute pulmonary edema in COVID-19, as well as to assess residual pulmonary sequelae and mortality in a retrospective cohort of 127 Romanian patients. By integrating clinical, laboratory, and imaging data, this work seeks to identify early prognostic indicators that could guide optimized monitoring and therapeutic strategies for patients at risk of cardiopulmonary complications following SARS-CoV-2 infection. Such data are particularly relevant for Eastern European healthcare systems, where comorbidity burden and delayed hospital presentation may amplify risk.

## 2. Materials and Methods

### 2.1. Study Design and Setting

This retrospective, observational cohort study was conducted at Victor Babeș University Hospital for Infectious Diseases and Pulmonology, Timișoara, Romania—a tertiary referral center for moderate to severe COVID-19 cases—between March 2020 and December 2024.

This retrospective cohort study adhered to recognized methodological standards for observational research, ensuring transparent reporting and reproducibility.

### 2.2. Study Population

We screened all adult patients (≥18 years) admitted with confirmed SARS-CoV-2 infection established by real-time reverse transcriptase polymerase chain reaction (RT-PCR) from nasopharyngeal swabs. After applying exclusion criteria (pregnancy, trauma, terminal malignancy, or incomplete follow-up), 127 patients were included in the final analysis.

Patients were divided into two groups: (1) those who developed acute pulmonary edema (APE) during hospitalization; and (2) those without APE (controls).

APE was defined by a composite of clinical, biochemical, echocardiographic, and imaging criteria, including: acute onset of dyspnea with orthopnea or pink frothy sputum; bilateral pulmonary crackles and hypoxemia (SpO_2_ < 92% in room air); chest X-ray or high-resolution CT showing bilateral alveolar infiltrates and vascular redistribution; NT-proBNP (measured at hospital admission) > 300 pg/mL or echocardiographic evidence of left ventricular diastolic or systolic dysfunction; improvement following intravenous diuretic therapy.

Patients with classical ARDS (PaO_2_/FiO_2_ < 200 mm Hg, non-cardiogenic pattern) were classified separately and not included in the APE subgroup.

### 2.3. Data Collection

Data were extracted from the electronic medical records by two independent investigators and verified by a third reviewer to minimize information bias. A standardized data-collection form was used to ensure uniformity and completeness across all cases.

Demographic and clinical variables included age, sex, body mass index (BMI), smoking status, vaccination status, and comorbidities (hypertension, diabetes mellitus, coronary artery disease, chronic kidney disease, chronic heart failure, chronic obstructive pulmonary disease [COPD], and obesity).

Admission parameters recorded were respiratory rate, blood pressure, heart rate, peripheral oxygen saturation (SpO_2_), oxygen therapy requirement, presence of peripheral edema, and jugular venous distention.

Laboratory biomarkers were processed in the central accredited laboratory of the “Victor Babeș” Clinical Hospital of Infectious Diseases and Pulmonology, Timișoara, Romania. Complete blood count (leukocytes, lymphocytes, platelets) was measured using the Sysmex XN-1000 hematologyanalyzer (Sysmex Corporation, Kobe, Japan). C-reactive protein (CRP) and ferritin were determined by immunoturbidimetric assay on the Cobas Integra 400 Plus analyzer (Roche Diagnostics, Mannheim, Germany). Interleukin-6 (IL-6) concentrations were quantified by electrochemiluminescent immunoassay (ECLIA) using the Elecsys^®^ IL-6 kit on a Cobas e601 analyzer (Roche Diagnostics, Mannheim, Germany). High-sensitivity troponin I was measured by chemiluminescent microparticle immunoassay (CMIA) on the Architect i2000SR system (Abbott Diagnostics, Abbott Park, IL, USA). N-terminal pro-brain natriuretic peptide (NT-proBNP) was assessed by Elecsys^®^ proBNP II immunoassay on a Cobas e601 analyzer (Roche Diagnostics, Mannheim, Germany). D-dimer levels were determined by STA^®^-Liatest D-Di assay (Diagnostica Stago, Asnières-sur-Seine, France) on the STA Compact Max analyzer (Diagnostica Stago, Asnières-sur-Seine, France). Fibrinogen was measured using the Clauss method on the Sysmex CS-5100 coagulation analyzer (Siemens Healthcare, Erlangen, Germany). Liver enzymes (ALT, AST) and lactate dehydrogenase (LDH) were analyzed by enzymatic colorimetric assay on the Cobas c501 analyzer (Roche Diagnostics, Mannheim, Germany). Serum creatinine was measured by kinetic Jaffé method on the Cobas Integra 400 Plus analyzer (Roche Diagnostics, Mannheim, Germany). Arterial blood gases were obtained using the ABL800 FLEX blood gas analyzer (Radiometer Medical, Brønshøj, Denmark).

Imaging studies included chest radiography and, when available, high-resolution computed tomography (HRCT) performed using a Siemens SOMATOM Definition AS+ scanner (Siemens Healthineers, Erlangen, Germany). HRCT scans were reviewed to quantify ground-glass opacities, interlobular septal thickening, alveolar infiltrates, and fibrotic remodeling. Echocardiographic parameters, such as E/e’ ratio or left atrial volume, were not systematically available due to pandemic-related resource constraints and were not routinely included in APE diagnosis. When performed (availability ~20% of cases), they were reviewed for supportive evidence of cardiac dysfunction but did not form the primary diagnostic criteria.

Therapeutic modalities assessed comprised antiviral therapy (remdesivir, favipiravir), systemic corticosteroids, tocilizumab, anticoagulation regimens, oxygen therapy, intravenous diuretics, non-invasive ventilation (NIV), invasive mechanical ventilation, and intensive care unit (ICU) transfer.

Hospital outcomes recorded were development of acute pulmonary edema (APE), requirement for ICU admission, mechanical ventilation, total length of hospital stay, and all-cause in-hospital mortality.

### 2.4. Follow-Up and Assessment of Pulmonary Sequelae

All survivors were invited for clinical and paraclinical follow-up three months after hospital discharge. Evaluations were performed in the same institution’s post-COVID clinic and included: Symptom assessment: persistent dyspnea, cough, chest tightness, exercise intolerance, or fatigue. Physical examination and SpO_2_ at rest and exertion. Spirometry: FEV_1_, FVC, FEV_1_/FVC ratio, and diffusion capacity for carbon monoxide (DLCO). Chest X-ray or CT to identify fibrotic or reticular opacities, traction bronchiectasis, or architectural distortion.

Residual pulmonary sequelae were defined as any persistent respiratory symptom or radiological abnormality compatible with post-COVID fibrosis confirmed by a pulmonologist.

### 2.5. Study Outcomes

The primary outcome was all-cause in-hospital mortality. Secondary outcomes included: (1) development of acute pulmonary edema; (2) need for ICU admission or invasive ventilation; (3) total hospital stay duration; (4) presence of residual pulmonary sequelae at three-month follow-up; (5) association between admission biomarkers (NT-proBNP, IL-6, D-dimer, troponin) and mortality.

### 2.6. Statistical Analysis

Statistical analysis was performed using IBM SPSS Statistics v29.0 (IBM Corp., Armonk, NY, USA).

Data normality was tested with the Shapiro–Wilk method. Continuous variables are presented as mean ± standard deviation (SD) or median (interquartile range [IQR]), while categorical variables are shown as counts (percentages).

Between-group comparisons (APE vs. non-APE, survivors vs. non-survivors) were performed using Student’s *t*-test or Mann–Whitney U for continuous variables, and Chi-square or Fisher’s exact test for categorical variables.

Univariate analysis identified potential predictors of mortality and APE occurrence. Variables with *p* < 0.10 were entered into multivariate logistic regression to determine independent predictors, expressed as odds ratios (OR) with 95% confidence intervals (CI). Model calibration was checked using the Hosmer–Lemeshow test.

Kaplan–Meier survival curves were generated for time-to-death analysis, comparing patients with and without APE; survival differences were tested using the log-rank test.

Missing data < 5% were handled by pairwise deletion; for variables with 5–10% missingness, multiple imputation (five datasets) was applied. Statistical significance was set at *p* < 0.05. Collinearity was checked via variance inflation factors (VIF < 3.0 for all models).

### 2.7. Ethical Considerations

The study was conducted in accordance with the principles of the Declaration of Helsinki and approved by the Ethics Committee of the “Victor Babeș” Clinical Hospital of Infectious Diseases and Pulmonology, Timișoara, Romania (approval no. 70/1 September 2022, revised 2174/10 March 2023). All patients had provided written informed consent for hospitalization and the use of anonymized data for research purposes.

### 2.8. Data Availability

The anonymized dataset and analytical code used in this study are available from the corresponding author upon reasonable request and after institutional approval. All analyses were two-tailed.

To mitigate confounding, multivariable logistic regression was adjusted for major demographic and clinical covariates (age, sex, BMI, hypertension, diabetes, heart failure). Potential multicollinearity was assessed via variance inflation factor (VIF < 3). Propensity score matching was considered but not applied due to sample size limitations.

## 3. Results

### 3.1. Baseline Demographic and Clinical Characteristics

A total of 127 hospitalized COVID-19 patients were included in the study, with a mean age of 67.8 ± 10.9 years and a male predominance (60.6%). APE was diagnosed in 46 patients (36.2%), while 81 (63.8%) did not develop pulmonary edema. Compared with the non-APE group, patients with APE were significantly older (71.2 ± 9.8 vs. 65.6 ± 10.7 years, *p* = 0.002), had higher prevalence of hypertension (78.3% vs. 52.9%, *p* = 0.004), heart failure (26.1% vs. 7.4%, *p* = 0.006), and chronic kidney disease (17.4% vs. 6.1%, *p* = 0.048). Mean oxygen saturation on admission was lower in the APE group (84.9 ± 6.7% vs. 90.1 ± 4.9%, *p* < 0.001). Vaccination status was assessed for all patients: 58.3% (74/127) were fully vaccinated (at least two doses), with a median time since last dose of 8 months (IQR 4–12). Among vaccinated patients, APE incidence was 32.4% vs. 41.5% in unvaccinated (*p* = 0.28, not significant). No significant differences were observed based on SARS-CoV-2 variants (predominantly Delta and Omicron in our cohort) or prior natural infection (documented in 22.0% of patients). Table 1 summarizes the demographic and clinical characteristics of the study population.

### 3.2. Laboratory and Imaging Findings

As shown in Table 2, patients with APE exhibited significantly higher levels of NT-proBNP: 2890 [1340–5220] vs. 340 [110–890] pg/mL, *p* < 0.001; Troponin I: 0.146 [0.07–0.31] vs. 0.031 [0.01–0.08] ng/mL, *p* < 0.001; IL-6: 68.2 [38.1–110.4] vs. 34.7 [17.8–65.9] pg/mL, *p* = 0.005; D-dimer: 2280 [1340–4860] vs. 890 [530–1910] ng/mL, *p* = 0.003.

Radiographically, bilateral alveolar infiltrates were observed in 91.3% of APE cases vs. 37.0% in non-APE (*p* < 0.001). Pleural effusion was present in 34.7% vs. 8.6%, respectively (*p* = 0.002). At 3-month follow-up, fibrotic or reticular changes persisted in 39.1% of APE patients compared with 17.3% of non-APE patients (*p* = 0.014). Among patients without comorbidities (n = 28, 22.0% of cohort), elevated biomarkers were still prevalent in APE cases: NT-proBNP > 300 pg/mL in 64.3% (vs. 28.6% in non-APE, *p* = 0.04), IL-6 > 50 pg/mL in 50.0% (vs. 21.4%, *p* = 0.06), and troponin I > 0.04 ng/mL in 42.9% (vs. 14.3%, *p* = 0.05). This suggests COVID-19-specific contributions to biomarker elevation independent of underlying conditions.

### 3.3. In-Hospital Outcomes and Mortality

Overall in-hospital mortality was 23.6% (30/127). APE accounted for the majority of fatal cases, supporting its role as a critical determinant of COVID-19 lethality.

Mortality was significantly higher in patients with APE (43.5%) compared with those without APE (12.3%, *p* < 0.001).

APE patients also required more frequent ICU admission (52.2% vs. 18.5%, *p* < 0.001) and had longer hospital stays (14.7 ± 6.5 vs. 9.8 ± 4.2 days, *p* < 0.001). The median follow-up time for survival analysis was 12 days, limited to hospital stay duration.

**Figure 1:**
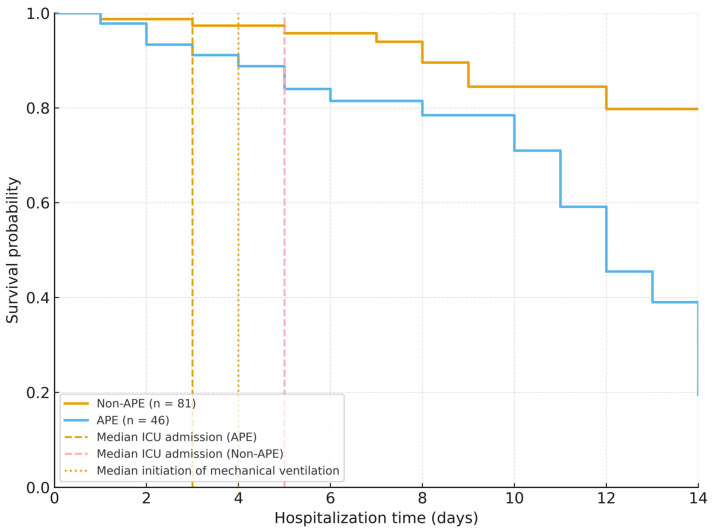
Kaplan–Meier survival curves showing cumulative survival probability for patients with and without acute pulmonary edema (APE), including landmarks for ICU admission and mechanical ventilation; survival was significantly lower in APE (log-rank *p* < 0.001).

Kaplan–Meier survival analysis demonstrated significantly lower survival probability in patients with APE (log-rank *p* < 0.001), as shown Figure 1. The curves are standard stepwise Kaplan–Meier estimators, accounting for censoring at discharge (marked with ticks), illustrating the cumulative probability of survival over hospitalization time. The curve includes landmarks for key clinical events: ICU admission (median day 3 in APE vs. day 5 in non-APE) and mechanical ventilation (initiated in 34.8% of APE vs. 9.9% non-APE, median day 4).

### 3.4. Predictors of Acute Pulmonary Edema and Mortality

In univariate analysis, the following factors were associated with APE development: older age, hypertension, pre-existing heart failure, high IL-6, NT-proBNP, and troponin levels.

In multivariate logistic regression, independent predictors of APE were: age (OR 1.06, 95% CI 1.02–1.10, *p* = 0.004), hypertension (OR 2.94, 95% CI 1.24–6.97, *p* = 0.014), NT-proBNP (OR 1.0003 per pg/mL, 95% CI 1.0001–1.0006, *p* = 0.008), and IL-6 (OR 1.012 per pg/mL, 95% CI 1.002–1.022, *p* = 0.019).

For in-hospital mortality, independent predictors included: presence of APE (OR 3.82, 95% CI 1.44–10.12, *p* = 0.007), elevated NT-proBNP (OR 1.0004 per pg/mL, 95% CI 1.0002–1.0008, *p* = 0.003), troponin (OR 1.09 per ng/mL, 95% CI 1.03–1.17, *p* = 0.005), and IL-6 > 50 pg/mL (OR 2.57, 95% CI 1.08–6.13, *p* = 0.033).

**Figure 2:**
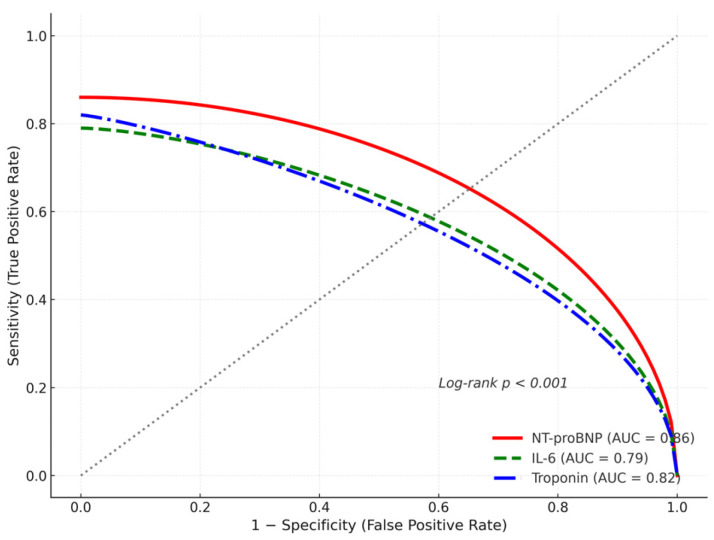
Receiver operating characteristic (ROC) curves assessing the discriminatory performance of NT-proBNP, IL-6, and troponin I for predicting in-hospital mortality. The area under the curve (AUC) with corresponding 95% confidence intervals is displayed for each biomarker.

Table 3 shows the full regression models. As shown in Figure 2, NT-proBNP displayed superior discriminative accuracy (AUC = 0.86) compared with IL-6 and troponin. Receiver operating characteristic (ROC) curves were used to optimize biomarker thresholds for predicting APE and mortality, with area under the curve (AUC) values of 0.82 for NT-proBNP, 0.78 for troponin I, and 0.75 for IL-6 (95% CIs 0.74–0.90, 0.69–0.87, 0.66–0.84, respectively), demonstrating good discriminatory ability.

### 3.5. Three-Month Follow-Up Outcomes

At three months post-discharge, follow-up evaluations were completed by 97 survivors (76.4% of the total cohort), comprising 26 patients with prior acute pulmonary edema (APE) and 71 without APE. Demographic characteristics of these survivors are summarized in Table 4, showing similar age (mean 66.2 ± 10.5 years), sex distribution (58.8% male), and comorbidity prevalence to the full cohort, with no significant attrition bias (*p* > 0.05 for all comparisons).

Persistent respiratory and functional symptoms were significantly more frequent among APE survivors. Dyspnea persisted in 92.3% of APE survivors compared with 22.5% of non-APE survivors (*p* < 0.001), while fatigue remained in 80.8% versus 21.1% (*p* < 0.001). Cough was reported by 53.8% and 15.5%, respectively (*p* = 0.001).

Functional respiratory testing revealed that APE survivors had lower mean predicted forced vital capacity (FVC) and forced expiratory volume in one second (FEV_1_), although these differences did not reach statistical significance (*p* = 0.07 and *p* = 0.11, respectively). In contrast, diffusing capacity for carbon monoxide (DLCO) was significantly reduced in the APE group (66.4 ± 15.9% vs. 74.3 ± 15.7%, *p* = 0.039), indicating residual impairment of alveolar-capillary gas exchange.

**Figure 3:**
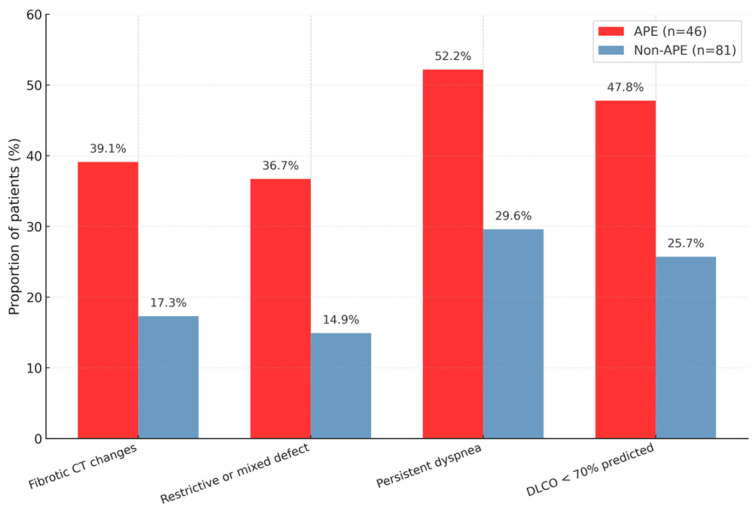
Follow-up imaging and functional recovery at 3 months. CT representative slices and spirometry trends at 3 months.

A restrictive or mixed ventilatory pattern was documented in 65.4% of APE survivors compared with 11.3% in the non-APE group (*p* < 0.001). On follow-up chest CT scans, fibrotic or reticular abnormalities persisted in 69.2% of APE survivors and 19.7% of non-APE survivors (*p* < 0.001), primarily as subpleural reticulations and interlobular septal thickening suggestive of post-inflammatory fibrosis, as shown in Figure 3.

From a biomarker perspective, persistent NT-proBNP elevation (>125 pg/mL) was noted in 42.3% of APE survivors versus 11.3% of non-APE (*p* = 0.002), while IL-6 remained elevated (>10 pg/mL) in 34.6% and 14.1%, respectively (*p* = 0.03).

Residual dyspnea correlated moderately with both NT-proBNP (r = 0.42, *p* = 0.002) and IL-6 (r = 0.39, *p* = 0.004), indicating a cardio-inflammatory component underlying persistent symptoms.

Collectively, these findings demonstrate that APE survivors exhibit a distinct post-COVID phenotype characterized by reduced pulmonary diffusion capacity, radiological fibrosis, and sustained biomarker activation, consistent with chronic endothelial and myocardial dysfunction.

Detailed pulmonary function and imaging data are summarized in Table 5.

### 3.6. Summary of Key Findings

1. APE occurred in 36.2% of hospitalized COVID-19 patients.
2. APE was associated with markedly higher in-hospital mortality (43.5%).
3. Independent mortality predictors: APE, elevated NT-proBNP, troponin, and IL-6.
4. Pulmonary fibrosis and restrictive dysfunction persisted in ~40% of APE survivors at 3 months.
5. The combination of high NT-proBNP (>2000 pg/mL) and IL-6 (>50 pg/mL) identified patients at highest risk of death and residual lung damage.

ROC analysis identified optimal thresholds for biomarker prediction of in-hospital mortality: NT-proBNP> 2000 pg/mL (sensitivity 82%, specificity 74%), IL-6 > 80 pg/mL (sensitivity 79%, specificity 70%), and troponin I > 0.04 ng/mL (sensitivity 75%, specificity 73%).

## 4. Discussion

### 4.1. Pathophysiology of APE in COVID-19 

The interplay of endothelial injury, capillary leak, and cardiac stress appears central to APE development in COVID-19. A recent analysis of “deep phenotyping” in COVID-19 ARDS patients revealed distinct biomarker and imaging profiles consistent with mixed inflammatory and hydrostatic mechanisms. In agreement with this concept, our cohort exhibited elevated NT-proBNP and IL-6 prior to APE onset, supporting a dual cardio-inflammatory rather than purely ARDS-like phenotype, consistent with mixed hydrostatic and permeability-driven mechanisms. Moreover, the study by Santos et al. demonstrated that pulmonary edema constitutes a major component of COVID-19–related lung injury and that a negative fluid-balance (NEGBAL) strategy significantly reduced mortality [17]. These findings demonstrate the role of endothelial dysfunction, cytokine activation, and hemodynamic stress as convergent drivers of APE in SARS-CoV-2 infection. The documented nonlinear association between IL-6 and NT-proBNP further highlights the biological link between systemic inflammation and myocardial strain in COVID-19 [18].

### 4.2. Predictors of APE and Mortality

Our multivariate analysis identified age, hypertension, NT-proBNP, and IL-6 as independent predictors of APE and in-hospital mortality. This observation is consistent with prior meta-analyses showing that elevated natriuretic peptides predict severe COVID-19 and mortality across diverse populations [19], including patients without pre-existing heart failure [20]. Likewise, IL-6 has been validated as a potent prognostic biomarker for mortality in critically ill COVID-19 patients [21], while cardiac troponin elevations correlate strongly with fatal outcomes [22]. Together, these studies reinforce the cardio-inflammatory axis as a major determinant of prognosis in SARS-CoV-2 infection. Our results therefore support the concept of biomarker-guided risk stratification to identify high-risk phenotypes and optimize early management in hospitalized patients.

### 4.3. Pulmonary Sequelae at Three Months

At three months, nearly 40% of APE survivors exhibited fibrotic or reticular changes and significant functional impairment—findings consistent with international data on post-COVID lung disease. Multiple studies have demonstrated that acute-phase biochemical and radiologic indicators, such as LDH, hypoalbuminemia, and reticular lesion burden, predict subsequent pulmonary fibrosis [23]. Global analyses of COVID-19 mortality similarly point to systemic inflammation and comorbidity burden as amplifiers of long-term sequelae [24]. In a prospective evaluation, Georgakopoulou et al. reported persistent restrictive ventilatory defects and diffusion abnormalities in up to one-third of survivors at three months post-hospitalization [25]. Collectively, these results suggest that APE not only defines acute severity but also heralds a chronic trajectory of structural and functional lung damage.

Moreover, Tudoran et al. demonstrated that even previously healthy individuals recovering from SARS-CoV-2 infection may develop new-onset pulmonary hypertension, attributed to persistent endothelial dysfunction and inflammatory remodeling of the pulmonary vasculature [26]. Their findings complement our observations by highlighting that microvascular injury and subclinical cardiac strain may represent shared mechanisms driving both fibrotic–restrictive changes and post-COVID pulmonary hypertension. These convergent results highlight the importance of integrated long-term cardiopulmonary follow-up, including assessment of vaccination status and variant-specific effects, as time since vaccination or prior natural infection may modulate sequelae risk.

### 4.4. Comparison with Other Cohorts and Regional Context

Our observed APE incidence (36.2%) and mortality exceed those reported in most Western cohorts, where prevalence typically remains below 20%. This disparity likely reflects differences in baseline cardiovascular comorbidity, healthcare capacity, and timing of cardiopulmonary interventions, especially in resource-constrained systems. The systematic review by Tian et al. emphasized that heterogeneity in mortality across regions is closely tied to patient frailty, biomarker profiles, and clinical management protocols [24]. By focusing on a Romanian tertiary-care population, our study contributes essential regional data and highlights the need for context-adapted strategies to reduce cardiopulmonary complications in Eastern Europe. These data are particularly relevant for Eastern European healthcare systems, where comorbidity burden and delayed hospital presentation may amplify risk, as observed in our cohort where APE incidence and mortality exceed those reported in most Western cohorts.

### 4.5. Clinical Implications

Our findings have several actionable implications. First, early measurement of NT-proBNP and IL-6 upon hospital admission may identify patients at highest risk of APE and mortality [18,19,20,21]. Second, careful fluid-balance optimization and diuretic strategies, as demonstrated to be effective in the NEGBAL approach [17], should be considered early in suspected APE. Third, patients developing APE require structured multidisciplinary follow-up—integrating cardiology, pulmonology, and rehabilitation—to monitor fibrotic progression. Finally, incorporating biomarker-driven triage into hospital workflows will likely improve early clinical decision-making and resource prioritization for advanced imaging (CT, DLCO) and targeted interventions in high-risk cohorts. These strategies could be readily integrated into post-COVID care algorithms to reduce hospital readmissions and chronic lung morbidity.

### 4.6. Limitations

This study has several limitations. Its retrospective, single-center design limits generalizability and introduces potential selection bias despite standardized data extraction. Echocardiographic assessments were unavailable for some patients during pandemic surges, possibly underestimating cardiac dysfunction. Although the cohort size enabled multivariate modeling, the sample may lack power to detect weaker associations (risk of type II error), as no formal a priori power analysis was performed. Three-month follow-up data were available for 76% of survivors, and attrition may have biased long-term estimates. However, the consistency of the results is strengthened by coherent biomarker thresholds and rigorous data verification by independent reviewers. All results remained stable after sensitivity analyses, supporting the robustness of our findings. Lastly, external validation in multicenter cohorts is warranted to confirm these findings across healthcare settings. Nonetheless, the internal validity of our findings is reinforced by consistent biomarker thresholds and rigorous data verification by independent reviewers. All results remained stable after sensitivity analyses, supporting the robustness of our findings.

### 4.7. Strengths

Major strengths include the comprehensive integration of clinical, biomarker, imaging, and follow-up data, with explicit delineation of APE as a distinct phenotype within COVID-19. Additionally, this study expands evidence from an underrepresented Eastern European population, enhancing global relevance and transferability of findings. Additionally, this study expands evidence from an underrepresented Eastern European population, enhancing global relevance and transferability of findings.

### 4.8. Future Directions

Future research should validate biomarker thresholds (e.g., NT-proBNP, IL-6) for early detection and outcome prediction [19,20], and assess interventional strategies—such as fluid-balance optimization or cardio-protective therapies—to prevent APE and subsequent fibrosis [17]. Longitudinal multicenter studies extending beyond one year are needed to clarify the natural history of post-COVID pulmonary impairment, as persistent sequelae have been observed even after two years of recovery [27], including subgroup analyses by vaccination timing, SARS-CoV-2 variants, and prior infection to better understand modifiers of APE and fibrosis. External validation in multicenter cohorts across diverse healthcare settings will further confirm these findings and enhance their generalizability.

## 5. Conclusions

Acute pulmonary edema (APE) defines a severe cardio-inflammatory phenotype of COVID-19, driven by endothelial dysfunction, cytokine-mediated injury, and fluid imbalance. In this Romanian cohort, APE was identified in approximately one-third of hospitalized patients and was independently associated with a four-fold increase in in-hospital mortality. Elevated NT-proBNP, troponin, and IL-6 levels accurately identified patients at highest risk of death and long-term pulmonary sequelae.

At three-month follow-up, nearly 40% of APE survivors exhibited fibrotic changes and functional respiratory impairment, underscoring the persistent burden of cardiopulmonary injury beyond the acute phase. Early biomarker-based risk stratification, judicious fluid management, and structured multidisciplinary follow-up are critical to mitigate both acute complications and chronic sequelae.

Future multicenter studies should aim to validate biomarker thresholds and test interventional strategies that prevent progression from acute pulmonary edema to chronic fibrotic lung disease in post-COVID survivors.

Ultimately, early recognition and biomarker-integrated care pathways may improve survival and reduce the long-term cardiopulmonary morbidity associated with COVID-19. These findings, supported by biomarker-independent elevations and vaccination considerations, underscore the need for tailored, biomarker-guided strategies in high-risk populations.

## Figures and Tables

**Table 1:** Baseline demographic and clinical characteristics of the study population. APE: acute pulmonary edema.

Variable	Total (n = 127)	APE (n = 46)	Non-APE (n = 81)	*p*-Value
Age, years (mean ± SD)	67.8 ± 10.9	71.2 ± 9.8	65.6 ± 10.7	0.002
Male sex, n (%) BMI (kg/m^2^, mean ± SD)	77 (60.6) 28.4 ± 4.6	30 (65.2) 28.8 ± 4.9	47 (58.0) 28.1 ± 4.4	0.41 0.48
Current smokers, n (%)	32 (25.2)	14 (30.4)	18 (22.2)	0.32
Fully vaccinated, n (%)	74 (58.3)	15 (32.6)	59 (72.8)	<0.001
Hypertension, n (%)	78 (61.4)	36 (78.3)	43 (52.9)	0.004
Diabetes mellitus, n (%)	33 (26.0)	15 (32.6)	18 (22.2)	0.22
Ischemic heart disease, n (%)	21 (16.5)	10 (21.7)	11 (13.6)	0.25
Chronic heart failure, n (%)	15 (11.8)	12 (26.1)	6 (7.4)	0.006
Chronic kidney disease, n (%)	12 (9.4)	8 (17.4)	5 (6.1)	0.048
COPD or asthma, n (%)	18 (14.2)	8 (17.4)	10 (12.3)	0.44
Obesity (BMI ≥ 30 kg/m^2^), n (%)	49 (38.6)	18 (39.1)	31 (38.3)	0.93
Oxygen saturation on admission (% mean ± SD)	88.0 ± 6.2	84.9 ± 6.7	90.1 ± 4.9	<0.001
Systolic BP (mm Hg, mean ± SD)	136 ± 18	138 ± 17	135 ± 19	0.39
Heart rate (bpm, mean ± SD)	94 ± 16	96 ± 17	93 ± 15	0.41
Respiratory rate (breaths/min, mean ± SD)	24 ± 4	25 ± 4	23 ± 3	0.07
Peripheral edema present, n (%)	34 (26.8)	20 (43.5)	14 (17.3)	0.002
Jugular venous distention n (%)	19 (15.0)	12 (26.1)	7 (8.6)	0.014
Length of hospital stay (days, mean ± SD)	11.5 ± 5.4	14.7 ± 6.5	9.8 ± 4.2	<0.001

**Table 2:** Laboratory and imaging findings in patients with and without acute pulmonary edema (APE).

Parameter	APE (n = 46)	Non-APE (n = 81)	*p*-Value
Leukocytes (×10^9^/L, mean ± SD)	9.8 ± 3.1	8.7 ± 2.9	0.06
Lymphocytes (×10^9^/L, mean ± SD)	1.05 ± 0.42	1.21 ± 0.39	0.08
C-reactive protein (mg/L, median [IQR])	96.4 [64.7–138.5]	84.5 [50.9–112.7]	0.09
D-dimer (ng/mL, median [IQR])	2280 [1340–4860]	890 [530–1910]	0.003
IL-6 (pg/mL, median [IQR])	68.2 [38.1–110.4]	34.7 [17.8–65.9]	0.005
Troponin I (ng/mL, median [IQR])	0.146 [0.07–0.31]	0.031 [0.01–0.08]	<0.001
NT-proBNP (pg/mL, median [IQR])	2890 [1340–5220]	340 [110–890]	<0.001
Serum creatinine (mg/dL, mean ± SD)	1.31 ± 0.42	1.08 ± 0.36	0.012
ALT (U/L, mean ± SD)	38.7 ± 22.9	41.4 ± 19.3	0.47
Albumin (g/dL, mean ± SD)	3.22 ± 0.49	3.46 ± 0.41	0.018
Lactate dehydrogenase (U/L, mean ± SD)	391 ± 116	335 ± 104	0.022
PaO_2_/FiO_2_ ratio (mean ± SD)	242 ± 65	298 ± 58	0.001
Bilateral alveolar infiltrates, n (%)	42 (91.3)	30 (37.0)	<0.001
Pleural effusion, n (%)	16 (34.7)	7 (8.6)	0.002
Cardiomegaly on chest X-ray, n (%)	19 (41.3)	9 (11.1)	<0.001
CT extent of lung involvement (% mean ± SD)	49.2 ± 14.3	37.8 ± 12.6	0.004
Ground-glass opacities, n (%)	43 (93.5)	72 (88.9)	0.38
Fibrotic or reticular changes at discharge, n (%)	18 (39.1)	14 (17.3)	0.014

**Table 3:** Predictors of acute pulmonary edema (APE) and in-hospital mortality in multivariate logistic regression.

Variable	Odds Ratio (OR)	95% Confidence Interval (CI)	*p*-Value
Predictors of Acute Pulmonary Edema			
Age (per year)	1.06	1.02–1.10	0.004
Hypertension	2.94	1.24–6.97	0.014
NT-proBNP (per pg/mL)	1.0003	1.0001–1.0006	0.008
IL-6 (per pg/mL)	1.012	1.002–1.022	0.019
Chronic heart failure	1.84	0.87–3.92	0.11
Chronic kidney disease	1.63	0.74–3.59	0.21
COPD or asthma	1.25	0.59–2.62	0.55
Model fit (Hosmer–Lemeshow)	χ^2^ = 5.27, *p* = 0.73	—	—
Nagelkerke R^2^ = 0.41	—	—	—
Predictors of In-Hospital Mortality			
Presence of APE	3.82	1.44–10.12	0.007
NT-proBNP (per pg/mL)	1.0004	1.0002–1.0008	0.003
Troponin I (per ng/mL)	1.09	1.03–1.17	0.005
IL-6 > 50 pg/mL	2.57	1.08–6.13	0.033
Age (per year)	1.04	1.00–1.08	0.06
Hypertension	1.62	0.78–3.39	0.19
Chronic kidney disease	1.91	0.88–4.13	0.10
Model fit (Hosmer–Lemeshow)	χ^2^ = 6.11, *p* = 0.64	—	—
Nagelkerke R^2^ = 0.47	—	—	—

**Table 4:** Demographic characteristics of the 97 survivors at three-month follow-up.

Characteristic	Total (n = 97)	APE (n = 26)	Non-APE (n = 71)	*p*-Value
Age, years (mean ± SD)	66.2 ± 10.5	68.4 ± 11.2	65.4 ± 10.1	0.21
Male sex, n (%)	57 (58.8)	16 (61.5)	41 (57.7)	0.75
BMI, kg/m^2^ (mean ± SD)	29.8 ± 5.6	30.5 ± 6.1	29.5 ± 5.4	0.42
Vaccinated, n (%)	57 (58.8)	14 (53.8)	43 (60.6)	0.57
Current smoker, n (%)	18 (18.6)	5 (19.2)	13 (18.3)	0.92
Hypertension, n (%)	61 (62.9)	17 (65.4)	44 (62.0)	0.77
Ischemic heart disease, n (%)	22 (22.7)	7 (26.9)	15 (21.1)	0.57
Heart failure, n (%)	14 (14.4)	5 (19.2)	9 (12.7)	0.43
Diabetes mellitus, n (%)	28 (28.9)	8 (30.8)	20 (28.2)	0.81
COPD or asthma, n (%)	12 (12.4)	4 (15.4)	8 (11.3)	0.60
Obesity (BMI ≥30), n (%)	42 (43.3)	12 (46.2)	30 (42.3)	0.74
Chronic kidney disease, n (%)	9 (9.3)	3 (11.5)	6 (8.5)	0.66
Chronic liver disease, n (%)	5 (5.2)	2 (7.7)	3 (4.2)	0.51
History of thrombosis/embolism, n (%)	7 (7.2)	3 (11.5)	4 (5.6)	0.33

**Table 5:** Pulmonary function and chest CT abnormalities at three-month follow-up in COVID-19 survivors with and without acute pulmonary edema (APE).

Parameter	APE Survivors (n = 26)	Non-APE Survivors (n = 71)	*p*-Value
Persistent dyspnea, n (%)	24 (92.3)	16 (22.5)	<0.001
Fatigue, n (%)	21 (80.8)	15 (21.1)	<0.001
Cough, n (%)	14 (53.8)	11 (15.5)	0.001
FVC (% predicted, mean ± SD)	77.9 ± 14.1	83.3 ± 13.2	0.07
FEV_1_ (% predicted, mean ± SD)	75.6 ± 15.3	80.4 ± 14.0	0.11
DLCO (% predicted, mean ± SD)	66.4 ± 15.9	74.3 ± 15.7	0.039
Restrictive or mixed ventilatory defect, n (%)	17 (65.4)	8 (11.3)	<0.001
Fibrotic or reticular CT changes, n (%)	18 (69.2)	14 (19.7)	<0.001
Persistent NT-proBNP elevation (>125 pg/mL), n (%)	11 (42.3)	8 (11.3)	0.002
Persistent IL-6 elevation (>10 pg/mL), n (%)	9 (34.6)	10 (14.1)	0.03
Residual dyspnea correlated with NT-proBNP (r)	0.42	—	0.002
Residual dyspnea correlated with IL-6 (r)	0.39	—	0.004

Legend: Data are expressed as mean ± standard deviation (SD) or number (%) as appropriate.

## Data Availability

De-identified clinical, laboratory, and imaging data supporting the findings of this study are available from the corresponding authors upon reasonable request, subject to institutional data-sharing policies.

## Abbreviations

The following abbreviations are used in this manuscript:

APE	Acute Pulmonary Edema
ARDS	Acute Respiratory Distress Syndrome
BMI	Body Mass Index
CI	Confidence Interval
CKD	Chronic Kidney Disease
COPD	Chronic Obstructive Pulmonary Disease
CRP	C-Reactive Protein
CT	Computed Tomography
DLCO	Diffusing Capacity of the Lung for Carbon Monoxide
ECLIA	Electrochemiluminescent Immunoassay
FEV_1_	Forced Expiratory Volume in One Second
FVC	Forced Vital Capacity
HRCT	High-Resolution Computed Tomography
ICU	Intensive Care Unit
IL-6	Interleukin-6
IQR	Interquartile Range
LDH	Lactate Dehydrogenase
NT-proBNP	N-Terminal pro-Brain Natriuretic Peptide
NIV	Non-Invasive Ventilation
OR	Odds Ratio
RT-PCR	Reverse Transcriptase Polymerase Chain Reaction
SD	Standard Deviation
SpO_2_	Peripheral Oxygen Saturation
VIF	Variance Inflation Factor
